# Cross-Cultural Adaptation and Validation of the Persian Version of the M. D. Anderson Dysphagia Inventory

**DOI:** 10.1055/s-0043-1776725

**Published:** 2024-01-24

**Authors:** Fardin Sharifi, Zahra Sadat Qoreishi, Jalal Bakhtiyari, Abbas Ebadi, Mohammad Houshyari, Samira Azghandi

**Affiliations:** 1 Speech Therapy Department, University of Social Welfare and Rehabilitation Science, Tehran, Iran; 2 Speech Therapy Department, Tehran University of Medical Sciences, Tehran, Iran; 3 Nursing Department, Baqiyatallah University of Medical Sciences, Tehran, Iran; 4 Radio Oncology Department, Shahid Beheshti University of Medical Sciences, Tehran, Iran

**Keywords:** M. D. Anderson dysphagia inventory, dysphagia, head and neck cancer, quality of life

## Abstract

**Introduction** Dysphagia is a common issue in patients with head and neck cancer (HNC) and is known to negatively impact their quality of life. To evaluate the impact of dysphagia on the quality of life of HNC patients, the M. D. Anderson Dysphagia Inventory (MDADI) questionnaire was developed.

**Objective** The present study aimed to culturally adapt and validate the MDADI for Persian-speaking individuals. The MDADI is a self-administered questionnaire designed to assess the impact of dysphagia on the quality of life of HNC patients.

**Methods** The original MDADI questionnaire was translated into Persian using the forward-backward method, following the guidelines of the World Health Organization (WHO) for cultural adaptation. The content validity of the Persian version, MDADI-P, was assessed by 10 speech-language pathologists using the content validity index (CVI). Seventy-five HNC patients completed the MDADI-P to evaluate its convergent validity, which was determined by comparing the results with the Short-Form 36 (SF-36) questionnaire. Internal consistency and test-retest reliability were assessed using Cronbach α coefficient and intraclass correlation (ICC), respectively.

**Results** The scale content validity index (S-CVI) for the MDADI-P was 0.90, indicating good content validity. The MDADI-P demonstrated satisfactory internal consistency (Cronbach α coefficient = 0.728) and test-retest reliability (ICC = 0.91). The total MDADI-P score exhibited a significant correlation with the physical and mental components of the SF-36 (0.456 and 0.349, respectively, *p* < 0.05).

**Conclusion** The findings of the present study confirm the suitability of the MDADI-P in terms of content validity, construct validity, internal consistency, and test-retest reliability.

## Introduction

Swallowing involves several steps in conveying a bolus from the oral cavity to the stomach, including the oral stage, the pharyngeal stage, and the esophageal stage. 1 Dysphagia occurs due to structural or neuromotor abnormalities related to these stages in the oral cavity, the oropharynx, the larynx, the hypopharynx, the velopharynx, and the upper esophageal sphincter. 2 3 Dysphagia, which can lead to problems such as pneumonia, aspiration, malnutrition, and even death, is a significant symptom in many neurological diseases such as stroke, multiple sclerosis, Parkinson disease (PD), dementia, and head and neck cancer (HNC). 1 4 Furthermore, this disorder can lead to long-term social and functional limitations, mood disorders such as anxiety and depression, nutritional deficiencies, and a decreased quality of life (QOL). 5 6 7

Head and neck cancer is a general term that encompasses cancer in the oral cavity, the larynx, the pharynx, the salivary glands, the thyroid, the nasal sinuses, and lymph nodes in the neck. 8 Dysphagia is a prevalent and critical condition in HNC patients, and various factors may contribute to its presence, including the primary tumor site and anticancer treatments. It can manifest before, during, and after radiotherapy. 9 10 Moreover, the clinical phenotypes of dysphagia (chewing difficulties, nasal regurgitation, oral retention of food bolus, and choking) and its complications (malnutrition and aspiration pneumonia) have a significant impact on the health and QOL of these patients. 8 10 The World Health Organization (WHO) defines QOL as “individuals' understanding of their position in life within their culture and value systems concerning their expectations, goals, concerns, and standards” 11 12 . To assess QOL in people with dysphagia, several questionnaires such as the Swallowing Quality of Life (SWAL-QOL), Dysphagia Handicap Index (DHI), and MD Anderson Dysphagia Inventory (MDADI) have been developed. The MDADI is specifically designed to assess QOL in patients with HNC. 13 The MDADI was first developed in the USA in 2001 and has since been translated into many other languages. 2 7 8 10 This tool is a widely used questionnaire for assessing patients' perspectives on their swallowing ability, the impact of dysphagia on their QOL, and the efficacy of dysphagia treatment. 2

The MDADI was originally developed in English and requires modification for use in other languages. Consequently, due to the lack of an available QOL questionnaire specifically designed for HNC patients in Iran, our objective was to translate, culturally adapt, and validate the MDADI in Persian. This process will render MDADI-P comprehensible and allow clinicians in Iran to easily administer it. Therefore, conducting this research is necessary and practical, as it can aid in the assessment of QOL in HNC patients with dysphagia.

## Materials and Methods

The present research was approved by the Human Participants' Ethics Committee (Reference number: IR.USWR.REC.1399.033).

## Participants and Data Collection

A cross-sectional study was conducted in three hospitals: Imam Hussein Hospital, Amir Aˈlam Hospital, and Shohadaye Tajrish Hospital in Tehran, Iran, between April 2020 and August 2020. Seventy-five patients were selected based on the inclusion criteria.

## Eligibility Criteria

The inclusion criteria were as follows: (1) age ≥ 18 years old, (2) histologically and pathologically confirmed HNC by a clinical oncology specialist, (3) diagnosed dysphagia by the researcher using the Northwestern Dysphagia Patient Check Sheet, and (4) willingness to participate in the study by signing informed consent.

The exclusion criteria were: (1) dysphagia due to other reasons and (2) impaired alertness.

## Patient-Reported Outcome Instruments

### MDADI

The MDADI is a psychometrically validated, self-administered, and reliable questionnaire developed by Chen et al. It aims to assess the impact of dysphagia on the health-related QOL in patients with HNC. It consists of 4 subdomains: global (1 question), emotional (6 questions), functional (5 questions), and physical (8 questions). 2 13 14 The global question measures the impact of swallowing ability on day-to-day activities. The emotional subdomain evaluates the affective reaction of HNC patients to swallowing problems. The functional subdomain illustrates how the daily activities of the patient are affected by the swallowing disorder, and the physical subdomain indicates the perception of the patient of the swallowing disorder. 9 10 15

The MDADI is scored on a 5-point Likert scale: 1. strongly agree; 2. agree;3. no opinion; 4. Disagree; and 5. firmly disagree. However, two items on the emotional and functional subdomains (E7: “I do not feel self-conscious when I eat” and F2: “I feel free to go out with my friends, neighbors, and relatives”) are reversely scored: 5. firmly agree; 4. Agree; 3. no opinion; 2. Disagree; and 1. strongly disagree. 2 The global domain is scored separately, while the average scores of other items in each subdomain are calculated. The average score is then multiplied by 20 to obtain a final score ranging from 20 (poor functioning) to 100 (high functioning). 2 8

### The Short Form-36 (SF-36)

The SF-36 is a general questionnaire for evaluating health-related quality of life (HRQOL). 14 It assesses eight subdomains and provides two summary scores (physical and mental). The eight subdomains include physical functioning (PF), bodily pain (BP), role limitations due to physical problems (RP), vitality (VT), general health perceptions (GH), role limitations due to emotional problems (RE), social functioning (SF), and mental health (MH). Higher scores indicate better HRQOL. 16 This tool was validated in Iran in 2005 by Montazeri et al. 17

### Top of Form

#### MDADI Translation Process

The MDADI translation process involves several steps that ensure accurate and culturally adapted translations based on WHO guidelines. Each step is carefully executed to maintain the integrity and quality of the translated questionnaire. 18

### Forward Translation

Initially, the author obtained permission from the creator. Then, the MDADI was translated by a translator and a speech-language pathologist (SLP). The focus during this translation was on conceptual accuracy rather than literal translation.

### Expert Panel

In this step, two SLPs who specialized in dysphagia were selected to identify and clarify any expressions or concepts within the translation. Their expertise ensured the accuracy and relevance of the translated content.

### Backtranslation

An independent translator (also an SLP), who was unfamiliar with the questionnaire, performed a backtranslation of the translated version into English. This backtranslation was sent to the creator for approval. Once approved, a pre-final Persian version of the MDADI was obtained for subsequent pretesting and cognitive interviews.

### Pretesting and Cognitive Interviewing

Before the final sampling, a pretest version of the MDADI was administered to 10 HNC patients with dysphagia under experimental conditions. The patients provided their feedback, comments, and suggestions regarding the inventory questions. Incorporating the valuable input of the patients, the final version of the questionnaire was prepared

### Final Version

The final version of the MDADI-P questionnaire was developed by diligently following all the aforementioned steps. Subsequently, the questionnaire retrieval steps were performed, ensuring content validity and data collection within the target population.

### Statistical Analysis:

Ten SLPs experienced in managing dysphagia evaluated the content validity of the MDADI-P. They assessed the relevance of items related to dysphagia, the quality of translation, fluency, and understandability. The content validity index (CVI) was calculated based on their scoring criteria.

Validity and reliability scores were calculated using IBM SPSS Statistics for Windows, version 23 (IBM Corp., Armonk, NY, USA). To determine reliability, 32 patients were reassessed using the MDADI-P questionnaire after 2 weeks. The interclass correlation coefficient (ICC) was used to evaluate the test-retest reliability, with ICC values > 0.7 considered acceptable. Values > 0.8 and 0.9 were regarded as indicating good and excellent reliability, respectively. 19 Pearson correlation coefficient was used to evaluate the correlations between continuous variables, while Spearman rho was used for ordinal variables. The internal consistency reliability of the combined MDADI score and each subdomain was assessed using Cronbach alpha tests. An internal consistency reliability level of at least 0.7 was considered acceptable.

## Results

### Sample Characteristics

Demographic data for the 75 patients (aged 20 to 88 years old) who participated in the study are presented in Table 1 . Most of the participants were male patients with an average age of 53 years old. The most common tumor sites were the oral cavity and the larynx, and the majority of patients received treatment through surgery and radiotherapy.

**Table 1:** Demographic and clinical characteristics ( *n* = 75)

Variable	Category	N (%)
Gender	Male Female	59 (78.7) 16 (21.3)
Age (years old)	Range Median Mean ± SD	20–88 57 53 ± 1.88
Site of lesion	Oral cavity Pharynx Larynx Others	21 (28) 15 (20) 21 (28) 18 (24)
Treatments	Surgery Radiotherapy Chemotherapy Surgery and radiotherapy Radiotherapy and chemotherapy Surgery and radiotherapy and chemotherapy None	7 (9.3) 14 (18.7) 4 (5.3) 20 (26.7) 15 (20) 9 (12) 6 (8)

### Content Validity

Ten SLPs scored most of the MDADI questions with a kappa coefficient ≥ 0.79, indicating excellent content validity. Additionally, two questions of the MDADI-P achieved a kappa coefficient of 0.66, demonstrating good content validity. Ultimately, we concluded that the MDADI-P, with an S-CVI of 0.9, has excellent content validity ( Table 2 ).

**Table 2:** Kappa coefficient score of the MDADI questions

QUESTION	Subscale	Kappa (CI)	Result
**1** **2** **3** **4** **5** **6** **7** **8** **9** **10** **11** **12** **13** **14** **15** **16** **17** **18** **19** **20**	Global Emotional Functional Physical Emotional Emotional Physical Emotional Functional Physical Physical Emotional Physical Functional Functional Physical Physical Emotional Physical Functional	1 0.9 0.66 0.79 0.79 1 1 0.79 0.79 1 1 0.9 0.9 1 0.9 1 1 1 0.79 0.66	Excellent Excellent Good Excellent Excellent Excellent Excellent Excellent Excellent Excellent Excellent Excellent Excellent Excellent Excellent Excellent Excellent Excellent Excellent Good

### Reliability and Internal Consistency

The total Cronbach alpha obtained for the MDADI-P was 0.728. The test-retest reliability for each subdomain of the MDADI-P (global, emotional, functional, and physical) ranged from 0.56 to 0.912. Furthermore, the ICC of the test-retest reliability for the total score of the MDADI-P was > 0.91 (0.81-0.95), which is considered appropriate ( Table 3 ).

**Table 3:** Cronbach α coefficients and intra-class correlation (ICC) for the MDADI subscales

	Standard deviation	ICC	95%CI
Global Emotional Functional Physical Total	13.8 10.22 12.57 8.34 7.90	0.56 0.84 0.91 0.85 0.91	0.42-0.73 0.681-0.924 0.82-0.95 0.7-0.92 0.81-0.91

Abbreviations: CI, confidence interval; ICC, intraclass correlation

### Construct Validity

The correlations between the subdomains of the MDADI-P and the SF-36 subdomains were measured using the Spearman correlation coefficient. The eight subdomains of the SF-36 are physical functioning, physical role, bodily pain, emotional role, social functioning, vitality, and general health. The results are presented in Table 4 . The total score of the SF-36 correlated with the total score of the MDADI-P (r = 0.42; *p* < 0.05). The total score of the MDADI-P also showed a good correlation with the physical and mental components of the SF-36 (0.456 and 0.349, respectively, *p* < 0.05). Correlations between the MDADI-P total score and other SF-36 subscales such as physical functioning, vitality, bodily pain, general health, and mental health were calculated (r = 0.292 to 0.467; *p* < 0.05). Additionally, the subscales of the MDADI-P demonstrated a good correlation with the physical functioning and general health subscales of the SF-36 (r = 0.194 to 0.405 and 0.201 to 0.396, *p* < 0.05, respectively). However, the subscales of the MDADI-P showed a weak correlation with the emotional role and physical role subdomains of the SF-36.

**Table 4:** Construct validity (convergent validity): Spearman correlation coefficients between the subdomains of the MDADI and the subdomains of the SF-36

MDADI SF-36	Global	Emotional	Functional	Physical	Total
Physical functioning	0.194	0.367*	0.327*	0.405*	0.467*
Social functioning	- 0.1	0.05	0.09	0.223	0.156
Physical role	0.02	- 0.105	- 0.04	0.08	- 0.028
Emotional role	0.43	- 0.145	0.07-	0.225	0.013
Mental health	0.112	0.267*	0.285*	0.142	0.292*
Vitality	0.233*	0.166	0.172	0.209	0.233*
Body pain	0.040	0.196	0.222	0.295*	0.304*
General health	0.201	0.396*	0.361*	0.319*	0.455*
Physical components	0.222	0.332*	0.327*	0.413*	0.456*
Mental components	0.179	0.206	0.250*	0.359*	0.349*
Total	0.186	0.268*	0.297*	0.433*	0.427*

* *p* < 0.05.

## Discussion

We translated the MDADI into Persian and confirmed its validity and reliability. Seventy-five patients who thoroughly answered all the questionnaires participated in the present study; therefore, the feasibility can be considered reliable.

For other language versions (Dutch, Korean, Portuguese, Japanese, Chinese, and Swedish), the total Cronbach α coefficients range from 0.81 to 0.95 (in Persian, it is 0.728). 7 10 14 20 21 22 The Cronbach α coefficient of the Persian version was 0.728, which is comparable to the MDADI in other languages.

Regarding test-retest reliability, the ICC for the global subscale of MDADI-P was 0.56. The other language versions (Japanese, Korean, and Swedish) also showed low scores for the ICC of the global subscale. This could be attributed to the fact that the global subscale consists of a single item. The emotional subscale of other versions (Dutch, Korean, Portuguese, Japanese, Chinese, and Swedish) demonstrated ICC values ranging from 0.88 to 0.93, and the MDADI-P also exhibited a good level for this subscale (ICC = 0.84). Moreover, the functional (ICC = 0.91) and physical (ICC = 0.85) subscales of MDADI-P showed excellent and good levels, respectively. In comparison, the ICC values for these subscales in other versions (Dutch, Korean, Portuguese, Japanese, Chinese, and Swedish) ranged from 0.84 to 0.97 and from 0.84 to 0.96, respectively. Overall, the total ICC for MDADI-P demonstrated an excellent level (ICC = 0.91), similar to other language versions. 7 10 14 20 21 22

Regarding construct validity, the correlation between the MDADI-P subdomains and the SF-36 subdomains was reported. The correlation between the physical subdomains of the MDADI-P and physical functioning in SF-36 was 0.405, which is consistent with the original MDADI (r = 0.40). Similarly, the MDADI-P exhibited divergent validity for the emotional (0.367) and functional (0.327) subdomains, as observed in the original MDADI. 2

The correlations of all subscales of the MDADI-P with the mental and physical subdomains of SF-36 were weak to moderate, similar to the findings of the Swedish, Spanish, and Brazilian versions. 7 9 14 The correlations between the subscales of MDADI-P and the physical components of the SF-36 ranged from 0.222 to 0.413, while the correlations between the mental components of the MDADI-P ranged from 0.179 to 0.359. Additionally, similar to the Spanish versions of the MDADI, the correlations between the physical and mental subdomains of the SF-12 with the subscales of the MDADI were between 0.314 and 0.495, and 0.391 and 0.503, respectively. 9

After translation and cultural modification, the MDADI-P was validated. During this process, it was important to ensure that the MDADI-P was understandable and easily applicable for clinical experts. Our findings revealed that the MDADI-P has high content validity and face validity, making it an easy and efficient method in clinical settings. Furthermore, our results demonstrated strong test-retest reliability for the MDADI-P.

Analyzing the limitations of the present study, the data collection coincided with the outbreak of the Covid-19 epidemic, resulting in a reduction of the sample size from 100 patients to 75. Additionally, due to the limited sample size, it was not possible to conduct factor analysis to examine construct validity. Therefore, conducting research with a larger sample size would allow for the investigation of other psychometric features of MDADI, such as factor analysis to assess construct validity.

## Conclusion

The content validity, construct validity, reliability, and internal consistency outcomes of our study support the validity of the MDADI-P. Therefore, it is a suitable method for evaluating the QOL in Persian patients with HNC for both research and clinical purposes.
